# Cystatin B Involvement in Synapse Physiology of Rodent Brains and Human Cerebral Organoids

**DOI:** 10.3389/fnmol.2019.00195

**Published:** 2019-08-16

**Authors:** Eduardo Penna, Angela Cerciello, Angela Chambery, Rosita Russo, Filippo M. Cernilogar, Emilia Maria Pedone, Carla Perrone-Capano, Silvia Cappello, Rossella Di Giaimo, Marianna Crispino

**Affiliations:** ^1^Department of Biology, University of Naples Federico II, Naples, Italy; ^2^Department of Environmental, Biological and Pharmaceutical Sciences and Technologies, University of Campania “Luigi Vanvitelli”, Caserta, Italy; ^3^Division of Molecular Biology, Biomedical Center, Faculty of Medicine, LMU, Munich, Germany; ^4^Institute of Biostructures and Bioimaging, National Research Council (CNR), Naples, Italy; ^5^Department of Pharmacy, University of Naples Federico II, Naples, Italy; ^6^Institute of Genetics and Biophysics “Adriano Buzzati Traverso”, National Research Council (CNR), Naples, Italy; ^7^Department of Developmental Neurobiology, Max Planck Institute of Psychiatry, Munich, Germany

**Keywords:** CSTB, synaptosomes, EPM1A, cerebral organoids, synaptic plasticity, local protein synthesis

## Abstract

Cystatin B (CSTB) is a ubiquitous protein belonging to a superfamily of protease inhibitors. CSTB may play a critical role in brain physiology because its mutations cause progressive myoclonic epilepsy-1A (EPM1A), the most common form of progressive myoclonic epilepsy. However, the molecular mechanisms underlying the role of CSTB in the central nervous system (CNS) are largely unknown. To investigate the possible involvement of CSTB in the synaptic plasticity, we analyzed its expression in synaptosomes as a model system in studying the physiology of the synaptic regions of the CNS. We found that CSTB is not only present in the synaptosomes isolated from rat and mouse brain cortex, but also secreted into the medium in a depolarization-controlled manner. In addition, using biorthogonal noncanonical amino acid tagging (BONCAT) procedure, we demonstrated, for the first time, that CSTB is locally synthesized in the synaptosomes. The synaptic localization of CSTB was confirmed in a human 3D model of cortical development, namely cerebral organoids. Altogether, these results suggest that CSTB may play a role in the brain plasticity and open a new perspective in studying the involvement of CSTB deregulation in neurodegenerative and neuropsychiatric diseases.

## Introduction

Cystatin B (CSTB) is an inhibitor of the cathepsin family of proteases, widely expressed in most cell types and tissues. In the central nervous system (CNS), CSTB has been detected in the dentate gyrus of the hippocampus and in the cerebellum. In particular, the cell type-specific expression of CSTB in developing cerebellum suggests that the expression of this protein is finely regulated (Riccio et al., 2005).

CSTB forms a complex with a number of proteins, some of which are specific to the CNS and exhibit a cytoskeletal localization such as neurofilament protein light chain (NF-L), and brain β-spectrin, suggesting that CSTB may have additional unknown role (Di Giaimo et al., 2002; Riccio et al., 2005). It has been proposed that CSTB is involved in the mechanisms preventing cerebral apoptosis (Pennacchio et al., 1998) and protecting neurons from oxidative stress. Consistent with this hypothesis, CSTB knock-out (KO) mice display signs of oxidative stress, progressive ataxia, and neuronal death (Lehtinen et al., 2009). Regulation of the redox state seems to be a crucial mechanism in which CSTB is involved. In fact, CSTB has a high capacity to interact with the superoxide dismutase 1 (SOD-1), a protein that has an important function in preventing the accumulation of reactive oxygen species (ROS; Ulbrich et al., 2014).

Mutations of CSTB cause progressive myoclonic epilepsy-1A (EPM1A), the most common form of progressive myoclonic epilepsy. EPM1A is an autosomal recessive disease, and the genotype of the patients most commonly attain an unstable dodecamer expansion repeat in the promoter region of the CSTB gene, which decreases the gene transcription. Interestingly, the severity of the disease is known to be inversely correlated with the amount of residual functional CSTB protein (Joensuu et al., 2008; O’Brien et al., 2017). Neuronal loss is a feature of CSTB-KO mice with GABAergic signaling pathway particularly affected (Joensuu et al., 2014). Accordingly, a significant reduction in VGAT labeling was observed both in the cortex of CSTB-KO mice and in one EPM1 patient, suggesting a critical role of altered GABA signaling in the development of the disease (Buzzi et al., 2012). Moreover, in the developing cerebellum of pre-symptomatic CSTB-KO mice expressions of several genes related to synaptic development and functions were altered, suggesting that CSTB may be implicated in synaptic plasticity (Joensuu et al., 2014).

CSTB was detected in the senile plaque of Alzheimer’s and Parkinson’s disease, suggesting its role in neurodegenerative diseases (Ii et al., 1993; Žerovnik, 2016). One of the common denominators in many neurodegenerative disorders is the deregulation of synaptic plasticity (Skaper et al., 2018). Indeed, the CNS responds to the injuries with rearrangement of the synaptic contacts, thus the mechanisms underlying synaptic plasticity may be important in understanding the etiopathology of neurodegenerative disease.

The cytoarchitecture of neuronal circuits is maintained “plastic” not only during embryonic development but also throughout adulthood. The ability of neurons to change themselves in response to the stimuli is accountable for the storage of new information into the brain, and for the ability of the organisms to adapt to the changing environment (Wefelmeyer et al., 2016). Neuronal plasticity is expressed not only as the physical rearrangements of subcellular compartments such as dendrites, axon and nerve endings but also by the modulation of the number and strength of synaptic connections. The synaptic plasticity requires rapid but subtle modulation of the proteome especially at the synaptic level, and, in this regard, local protein synthesis plays a key role in remodeling the synaptic regions in response to the external stimuli (Cagnetta et al., 2018). To date, a great deal of experimental data has demonstrated the importance of the local system of protein synthesis in the physiology of CNS not only during growth cone elongation and axonal outgrowth in the developing brain (Jung et al., 2014), but also for axonal maintenance in mature neurons (Shigeoka et al., 2016). In the CNS of adult rodents, presynaptic protein synthesis is modulated by learning (Eyman et al., 2007) and is known to be required for long term plasticity (Younts et al., 2016).

In this context, it is conceivable that CSTB makes an important contribution to synaptic plasticity. In this article, we demonstrated in adult rat brains the presence of CSTB in the synaptic region. We also showed that CSTB is secreted from synaptosomes in depolarizing conditions and locally synthesized in the synaptosomes. The synaptic localization of CSTB was confirmed also in mouse adult brain and interestingly, in a human 3D model of cortical development, namely cerebral organoids. The synaptic role of CSTB opens a new perspective in understanding the involvement of this protein in the plasticity of the brain and consequently in EPM1 etiology.

## Materials and Methods

### Animals

Male Wistar rats and C57BL/6 mice (Charles River, Calco, Lecco, Italy) of about 3 months of age were kept in animal house in a temperature-controlled room under a 12-h light-dark regimen (lights on at 6 AM) with food and water *ad libitum*. The animals were anesthetized by intra-peritoneal injection of chloral hydrate (40 mg/100 g body weight) and decapitated with a guillotine. The brain was removed, and brain cortex and cerebellum were quickly dissected and used immediately for synaptosomal preparation.

### Preparation of Synaptosomal Fraction

Synaptosomal fractions were prepared as previously described (Eyman et al., 2007). Briefly, cerebral cortex (Cx), and cerebellum (Cb) were homogenized in nine volumes of cold isotonic medium (HM: 0.32 M sucrose, 10 mM Tris-HCl, pH 7.4), using a Dounce homogenizer. After centrifugation of the homogenate (2,200 *g*, 1 min, 4°C), the sediment was resuspended in the same volume of HM and centrifuged under the same conditions to yield a washed sediment containing nuclei, cell debris, and other particulates (P1 fraction). The two supernatant fractions were mixed and centrifuged at a higher speed (22,000 *g*, 4 min, 4°C), to obtain a second sediment that was resuspended in the same volume of HM and centrifuged as described above. The washed sediment contained free mitochondria, synaptosomes, myelin, and microsomal fragments (P2 fraction). An aliquot of the P2 fraction (1 ml), with a protein concentration of 3.5 mg/ml was layered over a discontinuous gradient of 5% and 13% Ficoll dissolved in HM (2 ml each) and centrifuged at 45,000 *g*, 45 min, 4°C. The purified synaptosomes were recovered at the interface between the two Ficoll layers, diluted with nine volumes HM and sedimented by centrifugation at 22,000 *g*, 20 min, 4°C. The sediment was homogenized in HM and used for subsequent analyses.

### Gel Electrophoresis and Western Blot Analysis

Aliquots of homogenate and synaptosomes were lysed in RIPA buffer (50 mM Tris-HCl pH 8.8, 150 mM NaCl, 0.1% SDS, 0.5% NP-40, 0.5% DOC; protease and phosphatase inhibitor cocktail, Sigma-Aldrich) and centrifuged in Eppendorf 5415C microcentrifuge at 14,000 rpm, 5 min, 4°C. Proteins (5 μg/lane, in Sample Buffer: 60 mM Tris-HCl pH 6.8, 10% Glycerol, 2% SDS, 100 mM DTT 0.1% bromophenol blue) were separated in 10%–15% SDS-polyacrylamide gel and transferred to PVDF membranes (Merck-Millipore). Western blot analysis was performed as previously reported (Chun et al., 2004; Volpicelli et al., 2019). Briefly, filters were blocked for 2 h at RT in 5% (w/v) non-fat milk in Tris-buffered saline Tween-20 (TBST; 0.1% Tween, 150 mM NaCl, 10 mM Tris-HCl, pH 7.5) and probed with the following antibodies: anti-CSTB (1:4,000, ABIN271833 Antibodies), anti-Synaptophysin (SYP; 1:400,000, AB9272, Merck-Millipore), anti-β-actin (1:2,000, 612656, BD Biosciences), and anti-SOD-1 (1:1,000, ADI-SOD-100, Enzo Life Sciences). After several washings in TBST, membranes were incubated with secondary antibody against rabbit (1:20,000, A0545, Sigma-Aldrich) or mouse (1:20,000, NA931, GE Healthcare) linked to horseradish peroxidase. Signals were visualized by chemiluminescence (ECL, Millipore) on autoradiographic film (Fujifilm X-Ray Film).

### Click-iT Reaction

The synaptosomal fraction was incubated in Ringer medium (90 mM NaCl, 3 mM KCl, 2 mM MgCl_2_, 1 mM CaCl_2_, 1 mM glucose, 100 mM sucrose, 30 mM Tris-HCl, pH 7.5) in presence or absence (negative control) of 50 μM L-Homopropargylglycine (HPG, Invitrogen). After 2 h incubation at 37°C the reaction was stopped by cooling the samples on ice. Synaptosomes were then collected by centrifugation at 190,000× *g*, 20 min, 4°C, and the pellet was resuspended in an appropriate volume of a buffer containing 1% SDS and 50 mM Tris-HCl pH 8. The protein concentration was determined by Pierce BCA (Thermo Scientific). Aliquots of 50 μL containing 200 μg of proteins were processed for Click-iT reaction, following the manufacturer’s instruction (Invitrogen). Briefly, 100 μL of Component A containing biotin azide (40 μM) and 10 μL of deionized water were added to the aliquot. After vortexing, the sample, 10 μL of Component B (CuSO_4_), 10 μL of reaction buffer 1 and 20 μL of reaction buffer 2 were added. All the samples are gently mixed on Roto-Torque for 20 min at RT. Proteins were precipitated as a pellet by methanol/chloroform precipitation and store at −20°C until use. Newly synthesized proteins were isolated using Dynabeads M-280 Streptavidin (Invitrogen). Aliquots of 50 μL of Dynabeads were washed in PBS pH 7.4. The protein pellet was resuspended in PBS containing 0.02% Tween 20, added to the beads and mixed gently on Roto-Torque for 30 min. The samples were exposed to DynaMags for few minutes to separate newly synthetized proteins, bound to Dynabeads, from the rest of the reaction mixture. After four washing using PBS pH 7.4 Dynabeads were boiled in 0.1% SDS for 5 min at 95°C and the newly-synthetized proteins were collected in sample buffer for western blot analysis.

### Secretion of Proteins From Synaptosomes

Synaptosomes were incubated in Ringer medium or in depolarizing medium (43 mM NaCl, 50 mM KCl, 2 mM MgCl_2_, 1 mM CaCl_2_, 1 mM glucose, 100 mM sucrose, 30 mM Tris-HCl, pH 7.5) to detect depolarization-induced secretion. The reaction was initiated by adding the synaptosomal fraction (300 μg/ml) to the incubation medium. Immediately after that, an aliquot of the incubation medium was transferred to ice to stop the reaction (sample at time 0). After 2 h incubation at 37°C, the reaction was stopped by cooling the sample on ice. Synaptosomes were collected by centrifugation at 190,000 *g*, 20 min, 4°C. Secreted proteins, present in the supernatant, were concentrated by Amicon Centrifugal Filter Devices with a cut-off of 10 kDa (Merck-Millipore) and the concentration was determined by Bradford assay (BIORAD). The synaptosomal proteins, retained in the pellet, were resuspended in RIPA Buffer. Aliquots of all samples in the sample buffer were separated in SDS-PAGE as previously described and visualized by Coomassie gel staining or Western blot; for Coomassie staining gel was placed in Coomassie solution (10% Acetic acid, 25% Isopropyl alcohol, 0.25% Coomassie brilliant blue R-250 SIGMA).

### Mass Spectrometry

#### Tryptic Digestion and Sample Preparation for MS/MS Analyses

All chemicals, tosyl phenylalanyl chloromethyl ketone (TPCK)-treated trypsin were from Sigma Chemical Company (Milan, Italy) unless otherwise stated. Acetonitrile (ACN; CH3CN), formic acid (FA) and water LC-MS grade were from Fisher Scientific Italia (Rodano, Milano).

Secretomes from incubation of synaptosomes in both Ringer (sample-R) and depolarizing media (sample-D) were precipitated with trichloroacetic acid (TCA). Briefly, 40 μL of ice-cold 100% TCA was added to 160 μL of samples (~50 μg) and the mixtures were incubated for 30 min in ice. Following centrifugation (15 min, 14,000 rpm, 4°C) supernatants were discarded and the protein pellets were washed once with cold diethyl-ether and then with cold acetone. The protein pellets were then dried at room temperature and reconstituted in 50 μL of 50 mM NH_4_HCO_3_ pH 8.0 containing 10% ACN.

Samples were then subjected to disulfide reduction with DTT 10 mM (1 h at 55°C) and alkylation with iodoacetamide 20 mM (20 min at room temperature in the dark). Enzymatic hydrolyzes were performed on reduced and alkylated samples by adding TPCK-treated trypsin with an enzyme/substrate (E/S) ratio of 1:50 (w/w) in two steps. Mixtures were then incubated at 37°C for 16 h and frozen for subsequent analyses (Russo et al., 2019).

#### High-Resolution nanoLC-Tandem Mass Spectrometry

Mass spectrometry analyses of tryptic digests (2 μg) were performed on a Q-Exactive Orbitrap mass spectrometer equipped with an EASY-Spray nanoelectrospray ion source (Thermo Fisher Scientific, Bremen, Germany) and coupled to a Thermo Fisher Scientific Dionex UltiMate 3000RSLC nano system (Thermo Fisher Scientific; Russo et al., 2017). The solvent composition was 0.1% FA in water (solvent A) and 0.1% FA in ACN (solvent B). Peptides were loaded on a trapping PepMap^TM^100 μCartridge Column C18 (300 μm× 0.5 cm, 5 μm, 100 Å) and desalted with solvent A for 3 min with at a flow rate of 10 μL/min. After trapping, eluted peptides were separated on an EASY-Spray analytical column (50 cm × 75 μm ID PepMap RSLC C18, 3 μm, 100 Å) and heated to 35°C, at a flow rate of 300 nL/min by using the following gradient: 4% B for 3 min, from 4% to 22% B in 50 min, from 22% to 35% B in 10 min, and from 35% to 90% B in 5 min. A washing (90% B for 5 min) and a re-equilibration (4% B for 15 min) step were always included at the end of the gradient. Eluting peptides were analyzed on the Q-Exactive mass spectrometer operating in positive polarity mode with a capillary temperature of 280°C and a potential of 1.9 kV applied to the capillary probe. Full MS survey scan resolution was set to 70,000 with an automatic gain control (AGC) target value of 3 × 10^6^ for a scan range of 375–1,500 m/z and maximum ion injection time (IT) of 100 ms. The mass (m/z) 445.12003 was used as lock mass. A data-dependent top five method was operated during which higher-energy collisional dissociation (HCD) spectra were obtained at 17,500 resolution with AGC target of 1 × 105 for a scan range of 200–2,000 m/z, maximum IT of 55 ms, 2 m/z isolation width and normalized collisional energy (NCE) of 27. Precursor ions targeted for HCD were dynamically excluded for 15 s. Full scans and Orbitrap MS/MS scans were acquired in profile mode, whereas ion trap mass spectra were acquired in centroid mode. Charge state recognition was enabled by excluding unassigned charge states.

#### Data Processing

The acquired raw files were analyzed with Proteome Discoverer 2.1 software (Thermo Fisher Scientific) using the SEQUEST HT search engine (Di Giuseppe et al., 2017).

The HCDMS/MS spectra were searched against the Rattus norvegicus database available in UniprotKB (8,018 reviewed entries, release 2018_01, 31-Jan-2018) assuming trypsin (Full) as digestion enzyme with two allowed number of missed cleavage sites and a minimum peptide length of six residues. The mass tolerances were set to 10 ppm and 0.02 Da for precursor and fragment ions, respectively. Carbamidomethylation (+57.021464 Da) of cysteine and oxidation of methionine (+15.995 Da) were set as static modification and dynamic modification, respectively. False discovery rates (FDRs) for peptide spectral matches (PSMs) were calculated and filtered using the Target Decoy PSM Validator Node in Proteome Discoverer.

The Target Decoy PSM Validator Node specifies the PSM confidences based on dynamic score-based thresholds. It calculates the node-dependent score thresholds needed to determine the FDRs, which are given as input parameters of the node. Target Decoy PSM Validator was run with the following settings: maximum Delta Cn of 0.05, a strict target FDR of 0.01, a relaxed target FDR of 0.05 and validation based on *q*-value. The Protein FDR Validator Node in Proteome Discoverer was used to classify protein identifications based on *q*-value. Proteins with a *q*-value below 0.01 were classified as high confidence identifications and proteins with a *q*-value of 0.01–0.05 were classified as medium confidence identifications. Only proteins identified with medium or high confidence were retained resulting in an overall FDR of 5%. Only proteins identified with a number of unique peptides greater than one were considered.

### Ontology Annotation

Proteins identified by Mass Spectrometry were assigned to Gene Ontology (GO) biological process groups (Annotation data set: GO biological process complete release 20190202) using the PANTHER Overrepresentation Test (release 20190308; Mi et al., 2017) with default parameters and in reference to the *Rattus norvegicus* genes as background.

### Cerebral Organoids

Induced pluripotent stem cells reprogrammed from human newborn foreskin fibroblasts (CRL-2522, ATCC; O’Neill et al., 2018; Klaus et al., 2019) were used to generate cerebral organoids as previously described (Lancaster et al., 2013; Lancaster and Knoblich, 2014). Organoids were kept in 10 cm dishes on an orbital shaker at 37°C, 5% CO_2_ and ambient oxygen level with medium changes every 3–4 days. Organoids were analyzed at 35 days, 60 days and 70 days after plating. For synaptosomal fraction purification, a pool of 20–40 organoids was collected by centrifugation (500× *g* for 10 min). Organoids were resuspended in HM and homogenized in a Dounce homogenizer with nine volumes of HM. The P2 crude synaptosomal fraction was prepared as described above. Homogenate and P2 fraction, resuspended in the sample buffer, were processed for western blot analysis as previously described. For immunostaining 16 μm sections of organoids were made using a cryotome. Immunostainings were performed as described previously (Cappello et al., 2013). Nuclei were visualized using 0.1 μg/ml 4,6-diamidino-2-phenylindole (DAPI, Sigma Aldrich). SYP antibody (AB9272, Merck-Millipore), doublecortin (DCX) antibody (AB2253, Millipore), and CSTB antibody (ABIN223204, Antibodies) were incubated at the dilution of 1:1,000, 1:1,000 and 1:400 respectively. Immunostained sections were analyzed using Leica laser-scanning microscopes.

### Statistical Analyses

All the statistical analyses were performed using GraphPad Prism 7 software. Data were expressed as mean ± SEM. Differences among groups were compared by ANOVA or *t*-test as indicated in the Figure legends. Differences were considered statistically significant at *p* < 0.05.

## Results

### Presence of Cystatin B in Synaptosomal Fraction From the Rodent Brains

**Figure 1:**
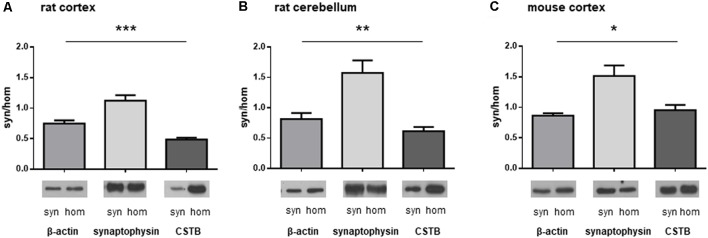
Differential distribution of cystatin B (CSTB), synaptophysin (SYP) and β-actin in the homogenate and synaptosomal fraction of rodent brains. Proteins obtained from homogenate and synaptosomes of rat and mouse brains were subjected to western blot analysis and the signals for CSTB, SYP and β-actin were quantified by densitometry; the signal ratio between synaptosomes (syn) and homogenate (hom) was plotted for each protein. **(A)** Homogenate and synaptosomal fraction from rat brain cortex. **(B)** Homogenate and synaptosomal fraction from rat cerebellum. **(C)** Homogenate and synaptosomal fraction from mouse brain cortex. Data are presented as means ± standard deviation (*n* = 4 rats, *n* = 3 mice). ANOVA statistical analysis indicated significantly different ratio syn/hom of each protein analyzed, *p < 0.05, **p < 0.01, ***p < 0.001.Representative images of the corresponding signals in the western blot were shown below each graph.

We isolated synaptosomal fractions from a homogenate of both cerebral cortex and cerebellum of rats as previously described (Eyman et al., 2007). By western blot analysis, we first assessed the distribution of a typical cytoskeletal protein, β-actin, in the homogenate and in synaptosomes. As shown in Figures 1A,B, β-actin was slightly less abundant in the synaptosomal fractions of both brain regions in comparison with its levels in the homogenates. By contrast, SYP, a well-known presynaptic protein, was significantly enriched in the synaptosomes of the brain cortex (Figure 1A) and cerebellum (Figure 1B). The differential distribution of these two proteins confirms that the synaptosomal fraction is a subcellular compartment representing the synaptic region of the neuron. When the distribution of CSTB in the synaptic compartment was examined (Figures 1A,B), it was evident that CSTB was present in rat synaptosomal fractions although it was more abundant in the homogenate, in keeping with its well-known cytosolic localization. The presence of CSTB in the synaptic region was also confirmed in the mouse cerebral cortex where the ratio of CSTB in synaptosomes vs. homogenate was even higher than in the rat (Figure 1C). Altogether, these results clearly indicate the synaptic localization of CSTB, suggesting its involvement in synaptic plasticity.

### Cystatin B Is Locally Synthesized in the Synaptosomal Fraction of Rat Brains

**Figure 2:**
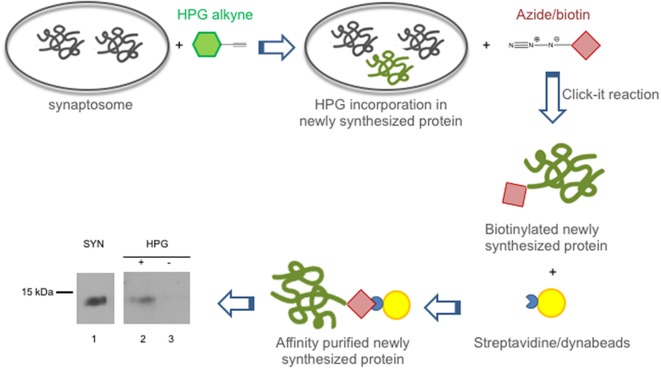
CSTB is locally synthesized in the synaptosomal fraction from rat cerebral cortex. Local synthesis of CSTB was assessed by metabolic labeling of synaptosomal proteins incubated with Homopropargylglycine (HPG) for 2 h as described in “Materials and Methods” section. The newly synthesized proteins, label with HPG (green), were biotinylated and isolated by affinity purification using streptavidin beads (yellow). Western blot analysis (representative image from two biological replicates), using CSTB antibody, was performed on synaptosomal proteins (SYN, lane 1), proteins newly synthesized in synaptosomal fraction (lane 2), negative control (incubation without HPG; lane 3). While 5 μg of proteins were loaded in lane 1, 50% of the pulldown was loaded in lanes 2 and 3 of the SDS gel for the western blot analysis.

In view of the crucial role played by synaptic protein synthesis in brain plasticity, we tested if CSTB was locally synthesized in the synaptosomal fraction. To this end, we performed metabolic labeling of newly synthesized proteins using Click-iT^TM^ L-HPG as a precursor (Best, 2009). Newly synthesized proteins incorporating HPG were isolated from the background proteins by biotinylation and subsequent purification with streptavidin beads. In Western blot analysis, CSTB was identified as one of the newly synthesized (HPG-labeled) proteins in the synaptosomes of rat cerebral cortex (Figure 2). The CSTB band was negligible in the negative control, i.e., the reaction mixture of the synaptosomal fraction incubated without HPG. These data demonstrated, for the first time, that CSTB is, at least in part, synthesized in the synaptic region of the rat cerebral cortex.

### Cystatin B Is Secreted by Synaptosomes

**Figure 3:**
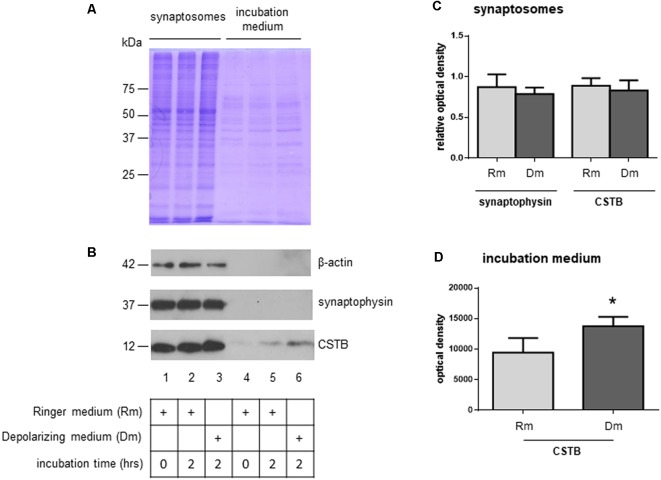
Secretion of CSTB from rat cortex synaptosomes. **(A)** Coomassie staining of proteins from synaptosomal pellet at incubation time 0 in Ringer medium (lane 1), incubation time 2 h in Ringer medium (lane 2), incubation time 2 h in depolarizing medium (lane 3); coomassie staining of proteins from incubation medium at time 0 (Ringer medium, lane 4), time 2 h (Ringer medium, lane 5) and time 2 h (depolarizing medium, lane 6). **(B)** Representative image of western blot analysis using β-actin, SYP and CSTB antibodies on the samples indicated in panel **(A)**. **(C)** The histograms shows the expression level of CSTB and SYP normalized to that of β-actin in synaptosomal pellet at incubation time 2 h in Ringer medium (lane 2 in panel **B**), incubation time 2 h in depolarizing medium (lane 3 in panel **B**) compared to incubation time 0 (lane 1 in panel **B**). Data are expressed as mean ± SEM from four independent experiments. **(D)** The histograms show the expression level of CSTB in Ringer incubation medium at time 2 h (lane 5 in panel **B**), and depolarizing medium at time 2 h (lane 6 in panel **B**). Data are expressed as mean ± SEM from three independent experiments. *Significantly different from Ringer medium by paired *t*-test (*p* < 0.05). Rm, Ringer medium; Dm, Depolarizing medium.

To investigate the role of CSTB in synaptic physiology, we tested its possible secretion from synaptosomes. Thus, the synaptosomal fraction was incubated for 2 h in physiological extracellular solution (Ringer medium). At the end of the incubation period, the incubation medium was collected, and its contents were concentrated using Amicon filters with a cut-off size of 10 kDa. As judged by the Coomassie blue staining, only few proteins were found secreted in the medium, with a pattern (Figure 3A, lane 5) that was strikingly different from that of synaptosomal proteins (Figure 3A, lane 2). Among the secreted proteins, we detected CSTB by Western blot analysis, while SYP, a very abundant protein of synaptic vesicles, was completely absent. Likewise, also β-actin was not present in the incubation medium (Figure 3B, lane 5).

As a next step, we checked if CSTB expression level was modulated by depolarization. Thus, synaptosomes were incubated for 2 h in a depolarizing medium containing high concentration of K^+^ (50 mM, see “Materials and Methods” section). The CSTB, as well as SYP expression levels in synaptosomes did not change in the two incubation conditions (Figure 3B, lanes 2 and 3, and Figure 3C). Interestingly, in depolarizing condition, the amount of secreted CSTB significantly increased compared with the incubation in physiological extracellular solution (Figure 3B, lanes 5 and 6 respectively, and Figure 3D), indicating that the secretion of this protein is regulated by depolarization. SYP, as well as β-actin were not detectable in the incubation medium in both experimental conditions in any of the biological replicates (Figure 3B, lanes 5 and 6).

### Mass Spectrometry of the Secreted Proteins

**Figure 4:**
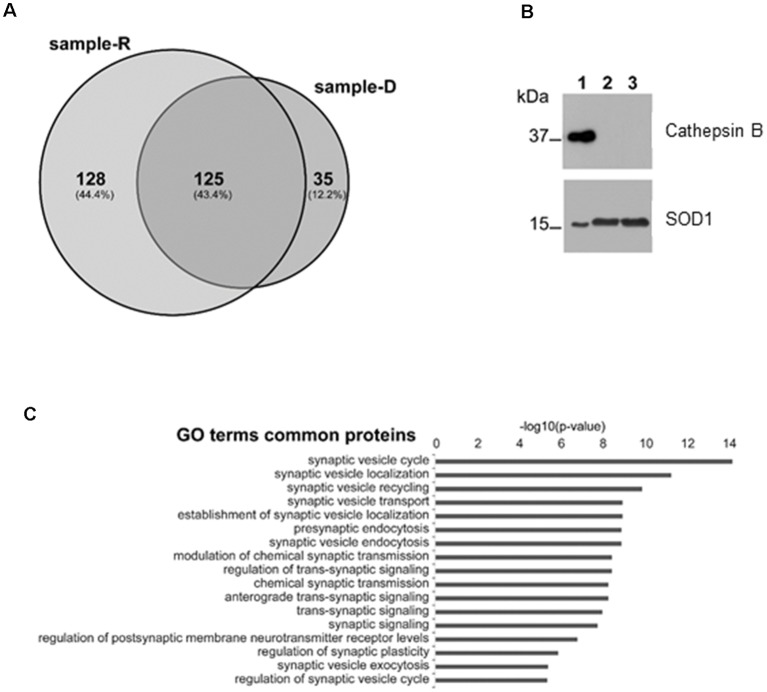
Mass spectrometry analysis of the proteins secreted by synaptosomes. **(A)** Venn diagram of the secreted proteins after 2 h incubation of the synaptosomes in Ringer medium (sample-R), and in the depolarizing medium (sample-D), identified by mass-spectrometry analysis. **(B)** Representative image of Western blot analysis using superoxide dismutase 1 (SOD1) and cathepsin B antibodies on proteins from synaptosomal fraction (lane 1), from Ringer incubation medium at time 2 h (lane 2) and from depolarizing medium at time 2 h (lane 3). The presence of SOD1 and the absence of cathepsin B in incubation media was verified, in four biological replicates, by Exact binomial test statistics. **(C)** Gene ontology (GO) analysis to classify the 125 common proteins based on biological processes determined by Panther (http://pantherdb.org), using Rattus norvegicus as reference species. Shown here are only terms related to synaptic activity with *p*-value < 1 × 10^−6^.

In order to identify the proteins secreted from synaptosomes and to survey the possible presence of known CSTB partners in the secreted medium, we performed a mass-spectrometry analysis of the secreted proteins after 2 h incubation of the synaptosomes either in Ringer medium (sample-R), or in the depolarizing medium (sample-D) by use of nanoLC-Tandem high resolution MS/MS. Using the filtering criteria indicated in “Materials and Methods” section, we detected 253 proteins in sample R and 160 in sample D, with 125 common proteins (Figure 4A; Supplementary Table S1). Among the proteins present in both samples, we identified some of the known partners of CSTB, such as Spectrin beta chain, Neurofilament light polypeptide (Di Giaimo et al., 2002) and SOD1 (Ulbrich et al., 2014), while we did not find any protease target of CSTB such as cathepsin B. The presence of SOD1 and the absence of cathepsin B in the secreted samples was validated by Western blot analysis (Figure 4B). As to the same set of common proteins, the results of GO analysis showed a strong enrichment for the terms related to the synaptic activity (Figure 4C), providing additional hint toward the involvement of CSTB in neuronal plasticity.

### Cystatin B Is Present in the Synaptic Regions of Human Cerebral Organoids

**Figure 5:**
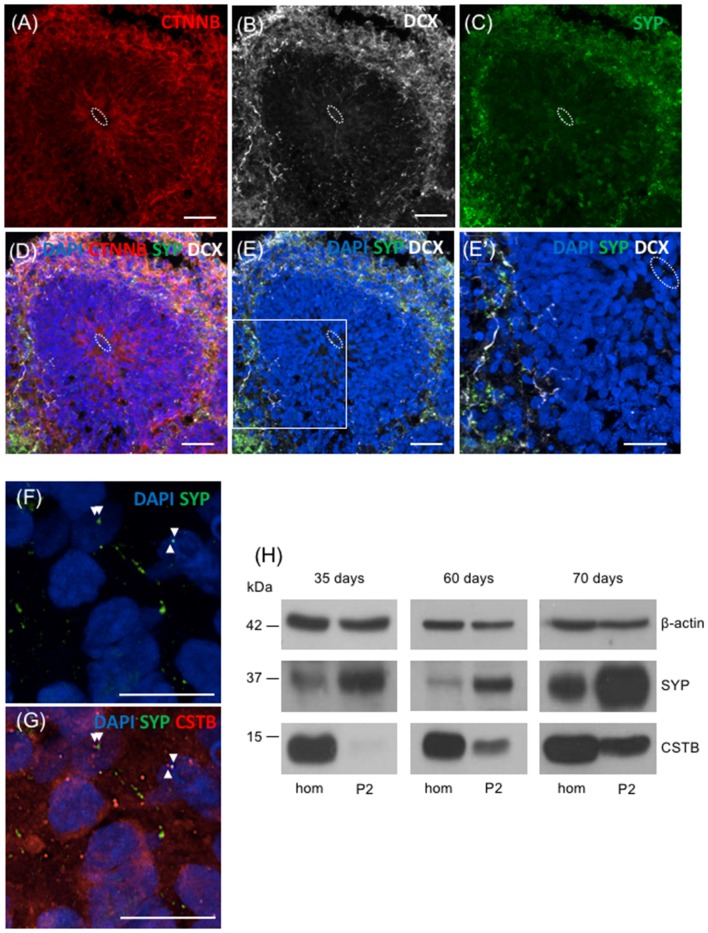
Presence of CSTB in the synaptic regions of cerebral human organoids. **(A–E’)** Confocal microscopic images of human brain organoids (70 days old) immunostained using antibodies against beta-catenin (CTNNB; red), doublecortin (DCX; white), and SYP (green). The cell nuclei were stained with 4,6-diamidino-2-phenylindole (DAPI; blue). In panels **(A–E’)** the white dashed circle indicates the ventricle-like area. **(E’)** Higher magnification of the boxed area in panel **(E)**. **(F,G)** Confocal microscopic images of sections of human brain organoids (70 days old) immunostained using antibodies against SYP (green), CSTB (red) and DAPI. The arrowheads in **(F,G)** indicate the colocalized (or very closely apposed) signals of SYP and CSTB. Scale bars, 30 μm in **(A–E)**, 20 μm in **(E’)**, 10 μm in **(F,G)**. **(H)** Western blot analysis of homogenate (hom) and crude synaptosomal fraction (P2) prepared from a pool of 20–40 cerebral human organoids 35 days, 60 days and 70 days old. Proteins (20 μg) from each fraction were loaded in the gel and the western blot analysis was performed using antibodies specific for β-actin, SYP, and CSTB.

To extend our study to humans, we took advantage of the 3D human multicellular model of brain cortical tissue, namely cerebral organoids (Lancaster et al., 2013; Lancaster and Knoblich, 2014; Camp et al., 2015). We first used immunohistochemical analyses to characterize the organoids. The ventricle like structures in the organoids were detected by immunostaining with beta-catenin (CTNNB) that labels adherent junctions allowing the identification of the apical belt, and with DCX that detects young neurons in the cortical plate-like zone (Figures 5A,B,D). To identify synaptic regions, 70 days old human brain organoids were stained with the presynaptic marker SYP (Figure 5C). As shown in Figures 5D,E,E′, the colocalization of DCX and SYP indicates that neurons in brain organoids developed in our laboratory express presynaptic markers to a certain extent, already at 70 days. In addition, we observed the localization of CSTB in some of the SYP positive-synaptic regions (Figures 5F,G). We prepared crude synaptosomal fraction (P2) from 35, 60 and 70 days old human brain organoids (pool of 20–40 organoids for each time point), and we analyzed the distribution of SYP, β-actin and CSTB in synaptosomes and in the corresponding homogenates (Figure 5H). SYP is highly enriched in the crude synaptosomal fraction compared to the homogenate, as expected for a pre-synaptic protein. On the other hand, as expected for a ubiquitous cytoskeletal protein, β-actin was equally distributed in the two fractions throughout the developmental stages of the organoids. These data are comparable to the ones obtained from rodent brains fractionation (Figure 1), indicating that synaptosomal fraction prepared from human brain organoids is a good model to study *in vitro* the synaptic region in a human model. CSTB has a different subcellular distribution than SYP and β-actin, being more abundant in homogenate than in synaptosomes (Figure 5H). These data indicate that in human brain organoids, as in rodent brains, CSTB is mainly a cytosolic protein, although it is partially localized also in the synaptic region. Interestingly, CSTB is almost undetectable in synaptosomal fraction from 35 days old organoids, while its level increases in synaptosomes at later stages, suggesting that the CSTB localization in the presynaptic terminals progressively increases with neuronal maturation.

## Discussion

In the present study, we demonstrated, for the first time, that CSTB is present in the synaptic region of the neuron. In particular, we found CSTB in the synaptosomal fractions of rat and mouse brain cortices. In addition, the presence of CSTB in the synaptosomes was also confirmed in human cerebral organoids. These data indicated that the synaptic location of CSTB is a conserved feature in different mammalian species, and suggested that this small ubiquitous protein may play an important role in brain physiology and plasticity. This hypothesis receives further support from the finding that CSTB is locally synthesized in the synaptic area. Indeed, pinpointing growing body of evidence has suggested that the synaptic system of protein synthesis serves as a crucial mechanism underlying the neuronal plasticity (Crispino et al., 2014; Younts et al., 2016; Cagnetta et al., 2019). Accordingly, its deregulation may contribute to the onset of several brain pathologies including neurodegenerative diseases (Baleriola and Hengst, 2015; Khalil et al., 2018).

To date, the precise mechanism by which CSTB may modulate synaptic plasticity is largely unknown, but several lines of studies have provided some interesting clues. The gene expression data obtained from the cerebellum of CSTB-deficient EPM1 mouse model, revealed that some of the genes involved in synaptic function and plasticity are deregulated already at the pre-symptomatic stage of the disease (Joensuu et al., 2014). This finding suggests that the presence or absence of CSTB may eventually exert its effect on neuronal plasticity by modulating gene expression. On the other hand, CSTB and its interactors were found in a multiprotein complex that included several proteins associated with neuronal physiology (Di Giaimo et al., 2002). Furthermore, the chaperon protein HSP70, which is affecting the folding state of CSTB (Rispoli et al., 2013), was reported to interact with the presynaptic protein syntaxin (Fei et al., 2007) and thereby participates in synapses remodeling (Karunanithi and Brown, 2015). In this context, our finding that CSTB is locally synthesized at the synaptic region add another layer of control by which the biological activity of CSTB is exquisitely modulated at the synapse.

Another novel finding we report in the present study is that CSTB is secreted by synaptosomes, and its secretion is enhanced by membrane depolarization. This finding suggests that CSTB secretion at the synapse is plastically regulated by neural activity, opening new scenarios for the role of this protein in the CNS. After secretion, what effect CSTB may exert to modulate synaptic plasticity would be an intriguing subject of future study. In this regard, it is tempting to speculate that CSTB, after secretion, could act as a neuroactive peptide or as a neurotrophic factor.

To identify the proteins secreted together with CSTB, we analyzed the synaptosomal incubation medium by mass spectrometry, and we detected several proteins known as binding partners of CSTB (Di Giaimo et al., 2002). Interestingly, although by MS we analyzed the same amount of total proteins for both physiological and depolarizing secretomes, fewer proteins were detected in depolarizing secretome. The two samples were overall similar by a technical point of view, as demonstrated by the comparable response of the base peak chromatograms, in terms of the relative intensity of ion signals. Therefore, we can speculate that the identification of fewer proteins in depolarizing medium by MS analysis could depend on effective less complex secretome. However, other factors affecting the total number of identified proteins, such as the different incubation media, cannot be excluded. By GO term enrichment analysis, the secreted proteins were grouped in numerous pathways, several of which were the ones involved in synaptic homeostasis. These results support our hypothesis that CSTB has a functional role in synaptic plasticity and that its deregulation may be part of the molecular synaptic mechanisms underlying the onset of neurodegenerative/neuropsychiatric diseases. Future experiments will address more in details the mechanism of CSTB secretion and the involvement of CSTB in plastic events of nervous system as for instance learning and memory.

An important finding of this work is the demonstration that CSTB is localized in the synaptic region also in a 3D human model of brain cortex, namely cerebral organoids. In terms of shape, size and complexity the human brain is quite distinct from the brains of other mammals commonly used as experimental model systems. In particular, the cerebral cortex is one of the brain regions that have developed most extensively throughout evolution, reaching the highest level of complexity in humans. Therefore, cerebral organoids provide the opportunity to study *in vitro* the complex interactions and functions of neuronal circuits, underlying unique features of human brain development and disease (Quadrato et al., 2017). The brain organoids may generate different type of cells with apicobasal polarity. Recently, single-cell RNA-sequencing analyses showed the expression of CSTB transcript in many cell types of human cerebral organoids (Velasco et al., 2019). Cerebral organoids can develop for more than 9 months, undergoing significant maturation, including generation of dendritic spines and the formation of spontaneously active neuronal network. Interestingly, our data in 70 days old cerebral organoids, showing some synapthophysin immunolabeling in young neurons (DCX-positive), indicate the presence of synapses already at this early stage of development in culture. Our results are in line with previous data reporting the presence of functional synapses in neurons from 180 days old organoids (Paşca et al., 2015). The crude synaptosomal fraction (P2), isolated for the first time from human cerebral organoids and characterized by the presence of SYP, is expected to provide invaluable insights into the molecular events taking place in the synaptic region of human brain cortex.

The presence of CSTB in the synaptic regions of human brain organoids, demonstrated by immunohistochemistry and western blot analysis on subcellular fractions, supports the hypothesis that this small protein contributes to neuronal plasticity also in humans.

More interestingly, the possibility to build cerebral organoids from the cells of human patients will facilitate *in vitro* modeling of neurodevelopmental and neuropsychiatric pathologies (Klaus et al., 2019), and thereby provide a new perspective to investigate the synaptic role of CSTB in the cellular and neural circuit dysfunctions related to neuropathologies.

## Data Availability

All datasets generated for this study are included in the manuscript and/or the Supplementary Files.

## Ethics Statement

This study was carried out in strict accordance with the Institutional Guidelines and complied with the Italian D.L. no. 116 of January 27, 1992 of Ministero della Salute and associated guidelines in the European Communities Council Directive of November 24, 1986 (86/609/ECC). All animal procedures reported herein were approved by the Institutional Animal Care and Use Committee (CSV) of University of Naples Federico II.

## Author Contributions

RDG and MC conceived and designed the experiments, analyzed the data, and wrote the manuscript. EP, ACe, ACh, RR and FC performed the experiments, collected the data, and performed the data analyses. EMP, CP-C and SC contributed to the discussion and to the editing of the manuscript.

## Conflict of Interest Statement

The authors declare that the research was conducted in the absence of any commercial or financial relationships that could be construed as a potential conflict of interest.
